# Vesicular glutamate transporters play a role in neuronal differentiation of cultured SVZ-derived neural precursor cells

**DOI:** 10.1371/journal.pone.0177069

**Published:** 2017-05-11

**Authors:** Eduardo H. Sánchez-Mendoza, Victor Bellver-Landete, Carmen Arce, Thorsten R. Doeppner, Dirk M. Hermann, María Jesús Oset-Gasque

**Affiliations:** 1 Department of Biochemistry and Molecular Biology, Faculty of Pharmacy, Complutense University of Madrid, Madrid, Spain; 2 Department of Neurology, University of Duisburg-Essen, Essen, Germany; 3 Department of Neurology, University of Göttingen Medical School, Göttingen, Germany; 4 Instituto Universitario de Investigación en Neuroquímica (IUIN), Universidad Complutense de Madrid (UCM), Madrid, Spain; Universidad de Castilla-La Mancha, SPAIN

## Abstract

The role of glutamate in the regulation of neurogenesis is well-established, but the role of vesicular glutamate transporters (VGLUTs) and excitatory amino acid transporters (EAATs) in controlling adult neurogenesis is unknown. Here we investigated the implication of VGLUTs in the differentiation of subventricular zone (SVZ)-derived neural precursor cells (NPCs). Our results show that NPCs express VGLUT1-3 and EAAT1-3 both at the mRNA and protein level. Their expression increases during differentiation closely associated with the expression of marker genes. In expression analyses we show that VGLUT1 and VGLUT2 are preferentially expressed by cultured SVZ-derived doublecortin+ neuroblasts, while VGLUT3 is found on GFAP+ glial cells. In cultured NPCs, inhibition of VGLUT by Evans Blue increased the mRNA level of neuronal markers doublecortin, B3T and MAP2, elevated the number of NPCs expressing doublecortin protein and promoted the number of cells with morphological appearance of branched neurons, suggesting that VGLUT function prevents neuronal differentiation of NPCs. This survival- and differentiation-promoting effect of Evans blue was corroborated by increased AKT phosphorylation and reduced MAPK phosphorylation. Thus, under physiological conditions, VGLUT1-3 inhibition, and thus decreased glutamate exocytosis, may promote neuronal differentiation of NPCs.

## Introduction

Glutamate plays key roles in the pathophysiology of cerebral ischemia and other neurodegenerative diseases [1–3]. Glutamate levels are regulated at the synaptic cleft by EAATs [4]. EAAT1 and EAAT2 are present in astrocytes, whereas EAAT3 and EAAT4 are located in neurons. Because of their biophysical properties, EAAT3 and EAAT4 could act as glutamate buffers by maintaining normal extracellular glutamate concentration, whereas excessive glutamate is withdrawn from the synapse by EAAT1 and EAAT2, thus preventing excitotoxicity [4].

In addition to the aforementioned mechanisms, glutamate concentrations are also regulated by modulating glutamate internalization into synaptic vesicles through VGLUTs 1, 2 and 3. VGLUT1 and VGLUT2 have a complementary distribution in the cortex and are also present in the caudate-putamen [5, 6]. VGLUT3 is found in the cortex and in the caudate-putamen among other structures, and it is less abundant than the other two isoforms [5]. Interestingly, VGLUTs are expressed by astrocytes *in vitro* [7]. Changes in VGLUT levels have been associated with several pathologies including schizophrenia, depression [8] or Parkinson’s disease [9].

We have previously proposed VGLUTs as possible pharmacological targets for stroke. We found that during early stages of reperfusion VGLUT1 is upregulated in the cortex (Cx) and striatum (St) whereas VGLUT2 and 3 are expressed by reactive glia in the ischemic corpus callosum (iCC) [10]. Interestingly, another work reported that stroke was associated with EAAT overexpression in glial cells within the iCC [11].

Adult neurogenesis has been clearly demonstrated in the subventricular zone (SVZ) of the lateral ventricles and the subgranular zone (SGZ) of the hippocampus, with controversial findings regarding the cortex [12]. Under physiological conditions stem cells in the SVZ proliferate and migrate towards the olfactory bulb (OB) forming chains of neuroblasts that are surrounded by glial cells along the rostral migratory stream (RMS) [13–15]. It is known that the interaction between neuroblasts and glial cells during the migration and differentiation process is regulated by numerous growth factors [16]. Nonetheless, recent evidence also suggests an important role for neurotransmitters in the regulation of neurogenesis in both health and pathology [17]. Indeed, glutamate and GABA balance have been proven to influence cell differentiation and survival in the RMS [18–22].

During the past decade the key role of neurotransmitters such as glutamate, in the regulation of neurogenesis and glial glutamatergic signaling has been clearly stated [19, 23–28]. NPCs express different types of glutamate receptors depending on their developmental stage. In fact, the presence of transcripts coding for various subunits of NMDA, AMPA, kainate receptors and group I, II and III metabotropic receptors, have been detected in neurospheres derived from embryonic cortex, along with those coding for GABA receptors [12]. Interestingly, blockade of the metabotropic glutamate receptor 5 (mGluR5) by genetic deletion or pharmacological interventions reduced the number of BrdU + cells along the iCC [29] whilst deletion of mGluR7 increased NPC proliferation but reduced neuronal differentiation [30]. However, nothing is known about the possible expression and role of vesicular or membrane glutamate transporters in adult neurogenesis under both, physiological and pathological conditions.

The well documented role of glutamate on NPC proliferation, migration and survival along the RMS under physiological conditions [18–21] together with the observations that VGLUTs and EAATs are expressed in glial cells in the iCC [10, 11] led us to hypothesize that VGLUT expression could play a direct role on the neuronal differentiation of SVZ-derived NPCs both in health and disease.

In this work, we have characterized the expression of VGLUTs and EAATs on SVZ stem cell cultures during different stages of differentiation and studied the functional implication of VGLUTs in neurogenesis by means of pharmacological inhibition with Evans Blue. Our data suggest a role of glutamate and its transporters in adult neurogenesis.

## Material and methods

All animal handling was performed in accordance with European Commission guidelines (2010/63/UE) and was approved by the Animal Research Committee at the Complutense University.

### SVZ-derived stem cell culture

NPCs were isolated from P0-3 rat pups as described by [16]. Briefly, rats were decapitated and the brains were removed and placed into cold DMEM/F12/Glutamax (GIBCO, Thermo Fisher Scientific, Spain). Meninges were removed and 300 μm coronal slices were produced with a custom-made tissue chopper. The SVZ was then isolated and treated with trypsin/EDTA (0.125%) during 10 min at 37°C with gentle shaking every 3 min. To stop the reaction, cells were centrifuged (1 min at 1000 rpm) and rinsed three times with DMEM/F12/Glutamax. Cells were then resuspended at 20,000 cell/mL in DMEM/F12/Glutamax containing EGF (GIBCO; 10 ng/mL). Cells were allowed to grow as neurospheres for 4–5 days before starting the experiments. For induction of cell differentiation required for subsequent Western blotting or Immunocytochemistry, plates and coverslips were treated with 50 μg/mL Poly-D-lysine (Sigma-Aldrich, Spain) diluted in sterile water overnight. Neurospheres were then seeded and allowed to attach for 10 min. Media containing growth factors was gently removed and immediately replaced with media without growth factors. Differentiation was carried out for a maximal period of 20 days. When necessary, the activity of VGLUTs was inhibited by addition of increasing concentrations of the dye Evans blue [31], a competitor inhibitor of VGLUTs, at final concentrations of 0.1, 1 and 5 μM for times between 3 to 5 days of differentiation. Samples were then prepared for western blotting or immunocytochemistry as indicated.

### Immunocytochemistry

Cells were fixed for 15 min with cold 4% paraformaldehyde in PBS. Washing steps were done 3 times with PBS 0.1% triton (PBS-T) before and after blocking and after incubation with primary and secondary antibodies. Blocking was carried out with 5% normal goat serum (NGS) or normal donkey serum (NDS) in PBS-T for 1 h depending on antibody combinations. Primary antibodies used were rabbit anti VGLUT1, rabbit anti VGLUT2 or rabbit anti VGLUT3 (Synaptic Systems, Germany, dilution 1:150), mouse anti GFAP (Sigma-Aldrich, dilution 1:250) or goat anti doublecortin (Santa Cruz Biotechnology, Spain, dilution 1:200). These antibodies were incubated overnight at 4°C in 1% serum in PBS-T. Secondary donkey anti rabbit alexa 488 or goat anti rabbit alexa 488 (Invitrogen, Thermo Fisher Scientific, Spain, dilution 1:250), biotinylated goat anti mouse (Invitrogen), or biotinylated donkey anti goat (Santa Cruz Biotechnology; dilution 1:500) were incubated for 1 h at room temperature (RT) in 1% serum PBS-T. For biotinylated antibody detection, an additional incubation step with streptavidin-texas red (Thermo Fisher 1:800) at RT in PBS-T was performed for 1 h. Cells where then washed 3 times with PBS and mounted on ProLong Gold Antifade with DAPI (Invitrogen). Preparations were allowed to dry for at least 24 h before performing detailed observation under the confocal microscope. Phenotype characterization of VGLUTs expression on DCX+ or GFAP+ cells at 3 and 7dd was performed by counting cells that expressed a VGLUT and either DCX or GFAP and normalized as percentage of the total cells present in a field as counted by total number of nuclei (DAPI+). At least 100 fields were counted in three different experiments at the mentioned time points.

### Morphological measurements

For morphological characterization of neuroblasts, goat anti doublecortin was incubated as detailed above and detected using biotinylated secondary donkey anti-goat (1:500) for 1 h at RT followed by 1 h incubation at RT with ABC complex (Vectastain, Vector labs, PALEX MEDICAL, S.A., Spain). After 3 washes in PBS, samples were incubated with 250 μl of PBS containing 500 μg/mL 3´3´diaminobenzidine and 0.01% H_2_O_2_ for 1 min. The reaction was stopped by repeated washes in PBS. Cells were then counterstained with hematoxylin for nuclear detection and dehydrated in 50% EtOH for 1 min and mounted on DePex mounting medium (Sigma). Images were acquired by bright field microscopy with a 40x objective for density and morphological characterization. Cells were classified as neuroblasts if they presented no clear axonal fiber (e.g. bipolar cells) when labelled with DCX, as compared to proto-neurons, which showed one specific axonal fiber with collateral protrusions characteristic of developing post-mitotic neurons on polarization stage 3–4[32] (S1 Fig). For axonal measurements, one fiber longer than 20 μM was selected. Length was quantified using the plug-in NeuronJ from ImageJ (NIH). A preliminary study on the suitability of DCX over MAP2 staining for morphological measurements was performed (S2 Fig).

### Microscopy

Light microscopy was performed on a Leica DM LB2 microscope and a digital Leica DFC 320 camera. Confocal microscopy was performed on a multispectral Leica TCS-SP2-AOBS confocal microscope (Leica Microsystems, Barcelona, Spain).

### Western blot analysis

Neurosphere cultures were homogenated in a protein extraction buffer containing 1% Triton, 10 mM Tris, 5 mM EDTA, 50 mM NaCl, 30 mM sodium pyrophosphate, 50 mM NaF, 100 μM orthovanadate, 500 μM PMSF, 5 μg/μL aprotinin and 0.025 μg/μL leupeptin, pH 7.4, and then centrifuged at 13,000 rpm at 4°C for 15 min. Supernatants were collected and stored at -80°C until used. The protein concentration was measured by the Bradford method (Quick Start Bradford Protein Assay; Bio-Rad, Spain) and 20 μg of protein were used for Western blot analysis. Western blots were carried out as previously described [10, 33]. Rabbit anti VGLUT1, rabbit anti VGLUT2 or rabbit anti VGLUT3 (Synaptic Systems, Göttingen, Germany); were used at a 1:2000 dilution. Guinea pig anti EAAT1, guinea pig anti EAAT2 or rabbit anti EAAT3 (Santa Cruz) were used at a 1:2000 dilution. Rabbit anti p-AKT (Ser473) or rabbit anti total AKT (Cell Signaling), rabbit anti MEK 1/2 and Rabbit anti p-MAPK(p44/42)(Thr202/Tyr204) (Cell Signaling), were used at a 1:2000 dilution. Mouse anti beta actin (Sigma) was used for loading control at a 1:2000 dilution. Band intensities were measured on a densitometric scanner, and normalized to β-actin expression. Quantification was performed using Image Gauge v. 4.0 (Science Lab. Fujifilm).

### RT-PCR

The RNeasy Mini Kit was used for total RNA isolation. Reverse transcription (RT) was carried out for 1 h at 55°C with oligodeoxythymidylate primer using 1 μg of total RNA from each sample for complementary DNA synthesis.

Real time quantitative PCR was performed in order to determine the mRNA levels of VGLUT and EAAT isoforms and housekeeping β-actin by using the following specific primers synthesized at Sigma-Aldrich Co. (Table 1):

**Table 1:** Primers used for RTqPCR.

	Gen	Primer 5'->3'		Product lenght	pb	%GC	Tm
***Glutamate Transporters***	***VGLUT1***	Fwd	GCTGTGTCATCTTCGTGAGG	110	20	55	52,99
		Rev	CAGCCGACTCCGTTCTAAGG		20	60	54,48
	***VGLUT2***	Fwd	TGGTGCAGTACACTGGATGG	123	20	55	53,82
		Rev	CGTCTGTTATGGTTGGATGC		20	50	51,23
	***VGLUT3***	Fwd	GCAATGACAAAGCACAAGACC	108	21	47,6	52,91
		Rev	TTCCCCAGAAGCAAAGACC		19	52,6	51,42
	***EAAT1***	Fwd	TGTCTTCTCCATGTGCTTCG	109	20	64,2	52,19
		Rev	CGCTACCAATCTCATGATGG		20	58,3	50,4
	***EAAT2***	Fwd	ATTGGTGCAGCCAGTAGGCC	100	20	50	63,8
		Rev	TTCTATCCAGCAGCCAGTCC		20	55	64,3
	***EAAT3***	Fwd	CAAGCGTGAAGAAGTGAAGC	137	20	50	52,1
		Rev	TGATGCCGTCTGAGTACAGG		20	55	53,39
***Neurogenesis*** ***Markers***	***DCX***	Fwd	AGGTAACGACCAAGACGCAAATGG	91	24	50	57,73
		Rev	AGGGCTTGTGGGTGTAGAGATAGG		24	54,17	57,17
	***NCAM***	Fwd	GCCGGCAGTTTACAATGCTGCG	112	22	59,09	59,87
		Rev	ACGCTGATTTCTCCTTGGCTGGG		23	56,62	59,19
	***Beta 3 tubulin***	Fwd	GTGAAGTCAGCATGAGGGAGATCG	119	24	54,17	57,16
		Rev	ATAGTTGCCGCTGGGGTCTATGCC		24	58,33	60,65
	***Nestin***	Fwd	TACATACAGGACTCTGCTGGAGGC	108	24	54,17	57,5
		Rev	AGGAAATTCGGCTTCAGCTTGGGG		24	54,17	59,46
	***GFAP***	Fwd	GCTCCAAGATGAAACCAACC	119	20	50	51,24
		Rev	CCAGCGACTCAACCTTCCT		20	57,89	53,53
	***SOX 2***	Fwd	ATCACAACAATCGCGGCGGC	70	20	60	58,96
		Rev	AGACGGGCGAAGTGCAATTGGG		22	59,09	59,99
	***TLX***	Fwd	TTCTTCACAGCGGTCACGCAGC	85	22	59,09	60,24
		Rev	TCACGAGAGTCTGCCGTTCAGG		22	59,09	58.35
***Control***	***rBACT***	Fwd	GCCAACCGTGAAAAGATGA	103	19	47,3	63,7
		Rev	TACGACCAGAGGCATACAGG		20	55	67,4

#### Real-time PCR

The SYBR Green PCR Master Mix (Applied Biosystems, Thermo Fisher Scientific, Spain) and the 7900 HT Fast Real-Time PCR system (Applied Biosystems) were used to detect the real-time quantitative PCR products of reverse-transcribed cDNA samples, according to the manufacturer's instructions. q-PCR conditions were: 95°C (10 min) followed by 40 cycles of 15 seconds at 95°C and annealing for 1 minute at 60°C. Three independent quantitative PCR assays were performed for each gene and measured in triplicate. Three no-template controls (NTCs) were run for each quantitative PCR assay, and genomic DNA contamination of total RNA was controlled using RT minus controls (samples without the reverse transcriptase).

### Statistics

Data were expressed as means±SEM values of three or four independent experiments with different cell batches, each one performed in triplicate. Statistical comparisons were performed by one-way analysis of variance (ANOVA) followed by Tukey post hoc test when analysis of variance was significant. Differences were regarded as significant for P <0.05. Statistical analyses were carried out using Sigmastat software (Systat Software, Inc., Germany).

## Results

### VGLUTs and EAATs are expressed at both the mRNA and protein level in SVZ-derived NPCs in primary culture in a time-dependent manner

**Fig 1:**
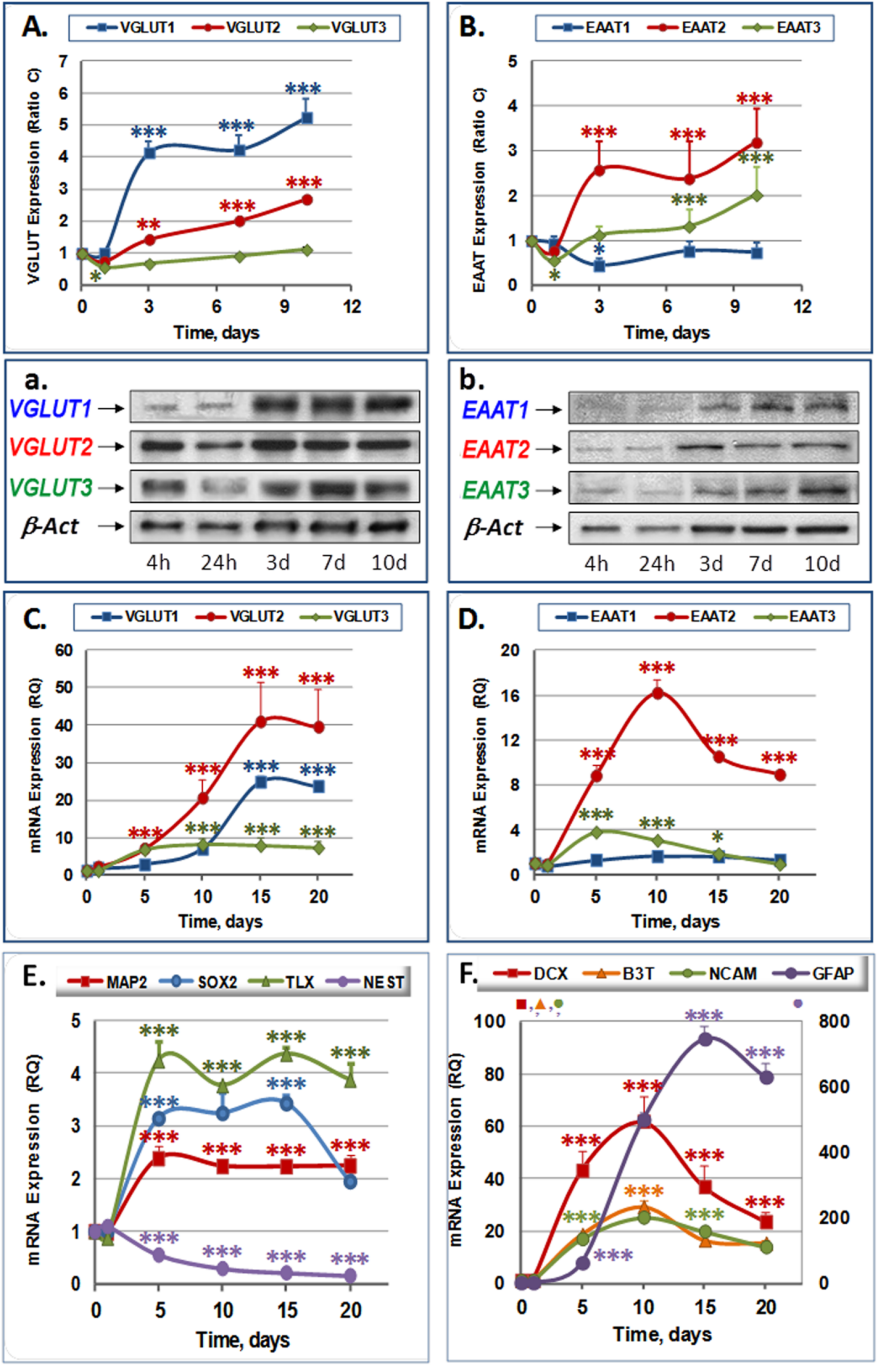
Time course evolution of glutamate transporters and differentiation markers in SVZ NPCs primary culture 1–20 days after differentiation. A-B) Time course evolution of of VGLUTs (E) and EAAT (F) protein expression in NPCs: Quantitative analysis. a-b) Representative images of Western blot experiments of VGLUT1-3 (a) and EAAT1-3 (b). C-D) Time course evolution of of VGLUTs (C) and EAAT (D) mRNA expression in NPCs E-F). Time course evolution of mRNA expression of specific neurogenic markers: Nestin, MAP2, SOX2 and TLX (E) and DCX, B3T, NCAM and GFAP (F). A clear reduction in Nestin mRNA expression is evident along with increased MAP2, SOX2, TLX (E) and DCX, B3T, NCAM, GFAP (F) mRNA expression. Western results were normalized to β-actin and are mean ± SEM of values obtained in four experiments, each one performed by duplicate. Statistics compare the expression at indicated times against control 4 h after seeding.* p<0.05; ** p<0.01; ***p<0.001; ANOVA test followed by Tukey post hoc test.

We first characterized the expression of VGLUTs and EAATs in SVZ cultures over 20 days by RT-qPCR. The analysis showed that VGLUT mRNAs are increasingly expressed during differentiation (Fig 1C). VGLUT1 mRNA increased by 6.88±0.88 fold at 10 days of differentiation (dd), reaching a maximum at 15 dd (24.78±1.73 fold). VGLUT2 mRNA was rapidly upregulated at 24 h after seeding (2.09±0.37 fold) and further increased until 15 dd (40.88±10.74 fold). VGLUT3 mRNA reached its maximum at 5–10 dd (8.01±1.75 fold) and remains stable thereafter (Fig 1C).

By contrast, EAATs showed a very different expression pattern. EAAT1 mRNA remained low throughout the time interval examined. EAAT3 mRNA increased between 5 dd and 10 dd (3.77±0.08 fold), but then declined to control levels. Remarkably, only EAAT2 mRNA steadily increased in a way comparable to VGLUT, peaking at 10 dd (16.17±1.22 fold) and dropping afterwards. However, it remained significantly higher than control levels (Fig 1D).

Accordingly, western blot analysis showed a gradual increase of VGLUTs protein levels. Thus, VGLUT1-3 protein levels increased between 3–10 dd (Fig 1A). VGLUT1 was already elevated at 3 dd reaching a peak between 7–10 dd (5.24±0.56 fold compared to control). VGLUT2 steadily increased until the end of the experiment reaching a maximum of 2.69±0.08 fold with respect to control. VGLUT3 was transiently downregulated at one day (0.56±0.02 fold) and recovered thereafter. The abundance of mRNA and protein of these transporters obviously correlated well with each other. Notably, VGLUT expression was detected from the beginning of the neurosphere differentiation, which could suggest glutamate might be loaded into vesicles during neurosphere development.

On the contrary, EAAT1 protein was transiently reduced at 3 dd, but then recovered to control levels at 7 dd (Fig 1B). EAAT2 protein increased at 3 dd, reaching maximum levels of 2.57±0.64 fold relative to control (Fig 1B). EAAT3 mildly increased at 3 dd, reaching maximum levels of 2.01±0.62 fold at 10 dd (Fig 1B).

#### Changes in mRNA expression of VGLUTs and EAATs in SVZ NPCs are significantly correlated with the mRNA levels of neurogenic markers

To validate VGLUT and EAAT expression with regard to NPC differentiation we also studied the expression of various differentiation markers. mRNA expression of the intermediate filament Nestin and the transcription factors SOX2 (SRY (sex determining region Y)-box 2) and TLX (orphan nuclear receptor subfamily 2 group E member 1 (NR2E1)), were chosen as indicators of the presence of proliferative NPCs. Nestin is highly expressed in proliferating cells and is progressively reduced as differentiation advances [17]. SOX2 activates the expression of the transcription factor Hairy and enhancer of split 5 (Hes5) [34], which is involved in cell proliferation thus retaining NPCs in a proliferative state. TLX, which is also induced by SOX2 in neurogenic areas, represses the expression of the tumor suppressing gene phosphatase and tensin homologue (PTEN) and various micro RNAs involved in cell differentiation, while promoting the expression of Wnt7a and other factors involved in NPC proliferation [35]. As indicators of neurodifferentiation, we chose the cytoskeleton proteins doublecortin (DCX), Beta-3-tubulin (B3T), MAP2 and neural cell adhesion molecule (NCAM). B3T is a component of the tubulin cytoskeleton that constructs the axon and presents an almost exclusively neuronal expression [36]. MAP2 is a microtubule associated protein highly enriched in primary dendrites [32] while DCX is microtubule associated protein characteristic of migrating neurons [17]. NCAM is a glycoprotein expressed in the cell surface whose levels of glycosylation are directly related to neuroblast migration [17]. Thus a reduction of Nestin, TLX and SOX2, opposite to an increase in DCX, B3T, NCAM and GFAP were considered as hallmarks of cellular differentiation. DCX, B3T, NCAM and GFAP mRNAs were markedly increased (61.67±9.48, 29.13±2.53, 25.12±0.93 and 498.06±25.41 fold, respectively) at 10 dd (Fig 1F). Likewise, MAP2, SOX2 and TLX mRNAs increased at 5 dd of culture (2.38±0.23, 3.14±0.03 and 4.24±0.38 fold, respectively) and remained high during the remaining days of culture, with the exception of SOX2 mRNA, which significantly decreased at 15dd (3.42±0.16) (Fig 1E). Nestin mRNA remained constant during the first 24 h of differentiation and then decreased at 5 dd (0.56±0.05 fold), remaining low until the end of the experiment (Fig 1E). Furthermore, analysis of linear correlation (Spearman Rank Order Correlation) between VGLUT and EAAT expression in relation with specific markers of the different phases of the neurogenesis process showed that significant positive lineal correlations exist between VGLUT1 and VGLUT2 mRNAs (S3A Fig) and between VGLUT3 and EAAT2 or EAAT1 mRNAs (S3K Fig), as well as between EAAT2 and EAAT1 mRNAs (not shown). In agreement with the RTqPCR data, a significant lineal correlation was found between VGLUT1 and VGLUT2 mRNAs and the markers Nestin (negative) and GFAP (positive) (S3B–S3E Fig), while a significant positive lineal correlation was found between VGLUT3 mRNA with DCX, B3T, NCAM and GFAP mRNAs (S3G–S3J Fig) and between EAAT2 (or EAAT1 to a lesser extent) with DCX, B3T and NCAM mRNAs (S3L–S3O Fig), but not with GFAP mRNA (not shown). EAAT3 mRNA showed a significant positive lineal correlation with DCX mRNA (r = +0.89; P<0.05; not shown). Thus, the expression of glutamate transporters is characteristic of the differentiation process of SVZ-derived NSCs and is associated with the expression of neuroblast and glial markers and negatively associated with the immature precursor cell marker nestin.

We observed that after 10 days of differentiation, the proliferation markers Nestin (which had been notably reduced), SOX2 and TLX remained largely stable. Meanwhile, the expression of the neuronal differentiation marker MAP2 did not increase to levels comparable to the neuroblast markers DCX and B3T, which abruptly decayed after this time thus indicating that neuroblasts in our conditions would remain in a state of proto-neurons or neuronal-like cells. Therefore time points below 10 days of differentiation were chosen for further western blot and immunocytochemistry analyses.

### VGLUT1 and 2 are preferentially expressed by neuroblasts whereas VGLUT3 is preferentially expressed by glial cells

Using immunocytochemistry we next examined the characteristics of cells expressing VGLUT proteins at 3 and 7 dd. These time points allow sufficient NPC differentiation as judged by DCX and GFAP expression (Fig 1F). Given that cells were seeded as neurospheres, neuroblasts and glial cells spread away from them during differentiation (S1A Fig). Therefore, VGLUT+ cells were found in the periphery of the neurospheres where DCX+ and GFAP+ expressing cells could be distinguished from one another. DCX+ neuroblasts and neuronal-like cells or “proto-neurons” could be distinguished based on the presence of one characteristic axonal-like projection containing clear collateral branches and one minor primary dendrite compared to a bipolar neuroblast in which no axonal-like process can be defined (S1B Fig).

**Fig 2:**
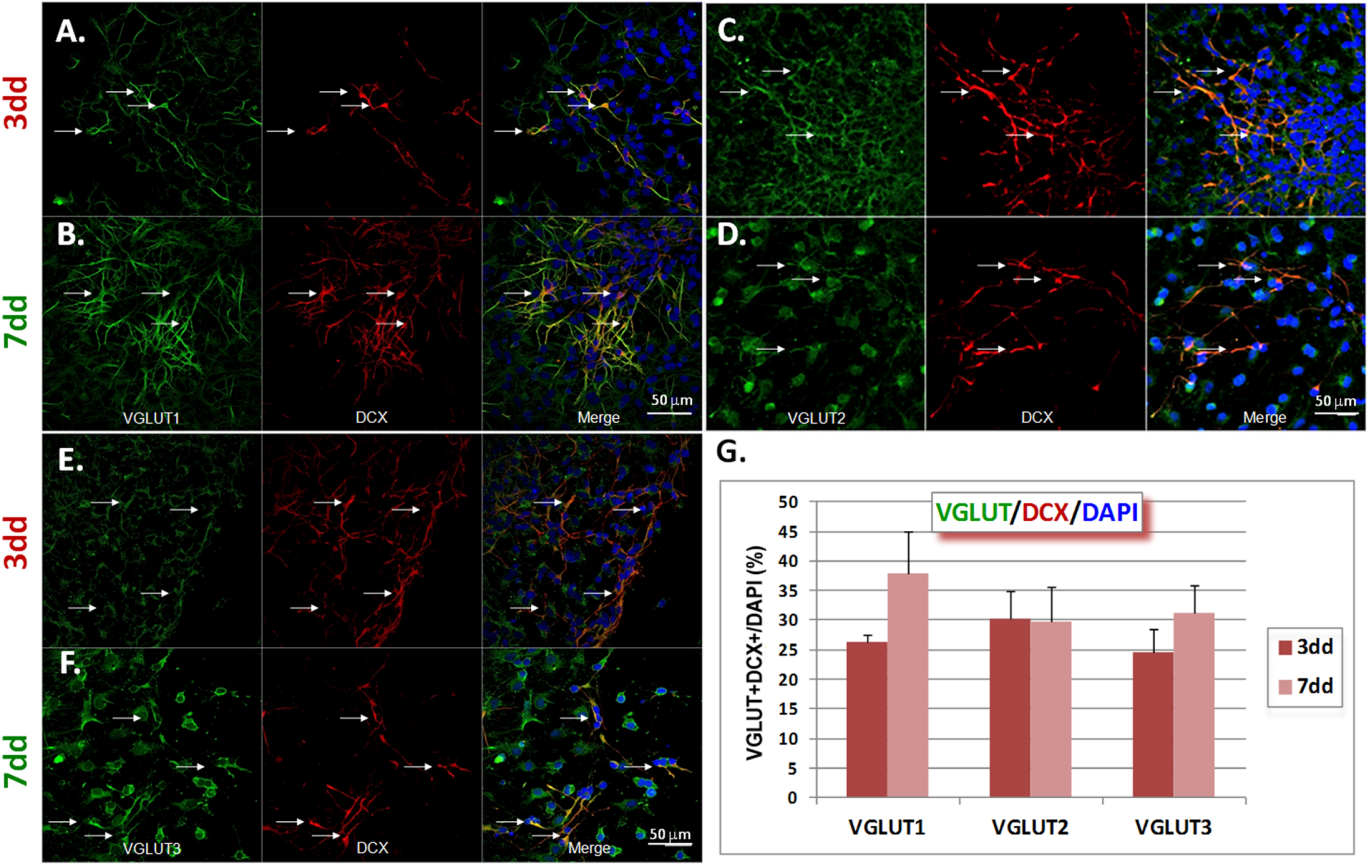
Co-expression of VGLUT1-3 and DCX in NPCs at 3 and 7 days of differentiation. A-B) VGLUT1, DCX and merge images at 3 (A) and 7 (B) days of differentiation, respectively. (C-D) VGLUT2. (E-F) VGLUT3. Quantification was performed by counting of double labelled cells with respect to the total number of cells in the field as determined by DAPI staining. Data are expressed as mean ± SEM. Statistical analysis was done by Student t-test. 150–200 cells per image were analyzed; n = 10 images from 2 different cultures. Scale bar = 50 μm.

**Fig 3:**
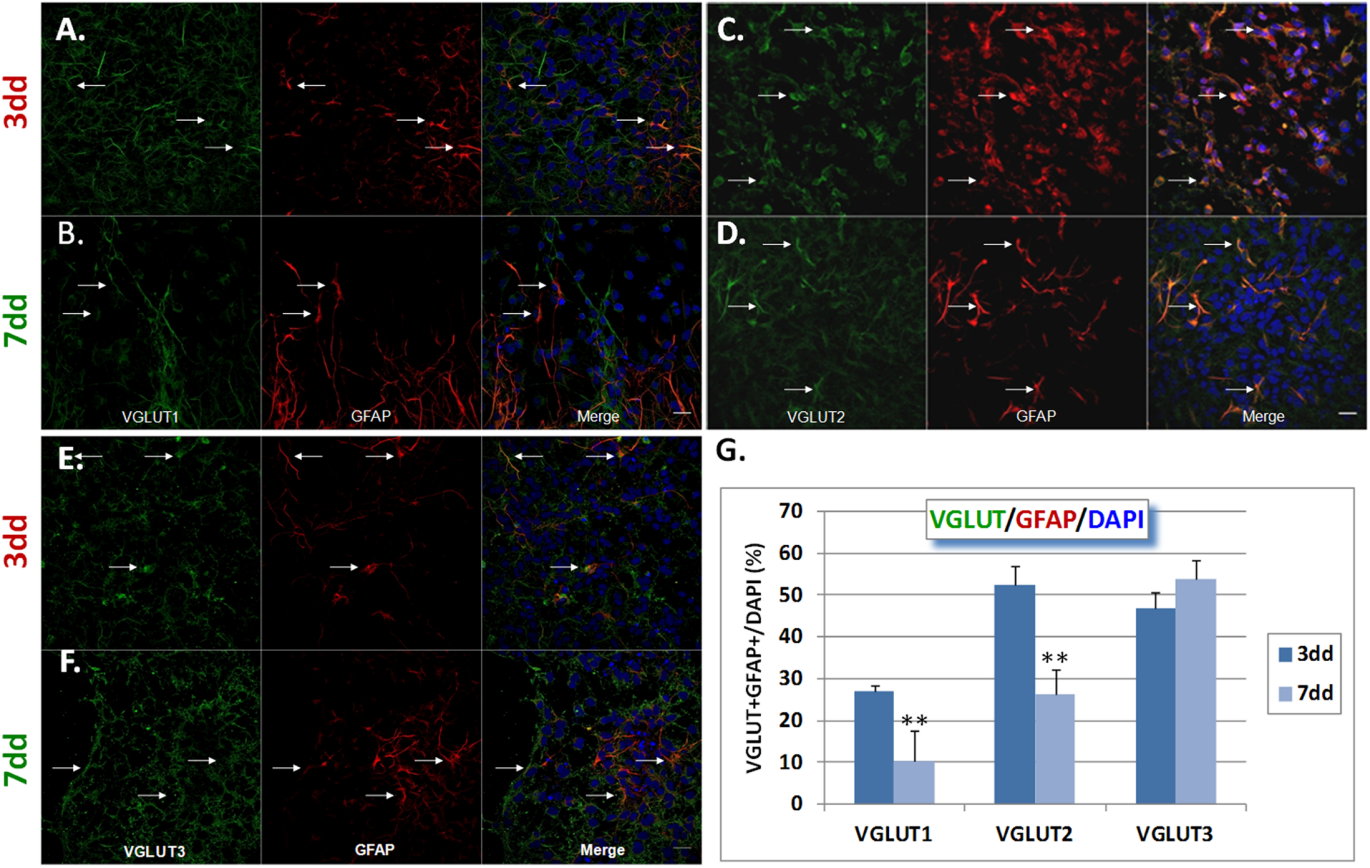
Co-expression of VGLUT1-3 and GFAP in NPCs at 3 and 7 days of differentiation. A-B) VGLUT1, GFAP and merge images at 3 and 7 days of differentiation, respectively. (C-D) VGLUT2. (E-F) VGLUT3. A statistically significant reduction in the number of GFAP cells expressing either VGLUT1 or 2 was found between 3 and 7 dd (** P<0.01), but not for VGLUT3. Quantification was performed by counting of double labelled cells with respect to the total number of cells in the field as determined by DAPI staining. Data are expressed as mean ± SEM. Statistical analysis was done by Student t-test. 150–200 cells per image were analyzed; n = 10 images from 2 different cultures. Scale bar = 50 μm.

VGLUTs 1–3 were found on neuroblasts and glial cells (Figs 2 and 3 and S4 Fig). Therefore, we decided to characterize the number of VGLUT+DCX+ or VGLUT+GFAP+ cells in proportion to the total number of cells per field as detected by DAPI counterstaining. The results indicate that VGLUT1+DCX+ cells tend to increase between 3 and 7 dd, although this increase was not statistically significant (26.17±1.47% to 37.81±7.23%, p = 0.167) (Fig 2A, 2B and 2G). VGLUT2+DCX+ cells remained constant (30.35±4.58% vs. 29.81±5.79%, p = 0.945) (Fig 2C, 2D and 2G), as were VGLUT3+DCX+ cells (24.51±4.07% to 31.10±4.81% (p = 0.346) (Fig 2E, 2F and 2G). By contrast, VGLUT1+GFAP+ and VGLUT2+GFAP+ cells decreased significantly between 3 and 7 dd (27.08±3.01% to 10.45±2.47%, p<0.01, and 52.38±4.88% to 26.44±3.86% p<0.01, for VGLUT 1 and 2, respectively) (Fig 3A–3D and 3G), whereas VGLUT3+GFAP+ cells remained constant (46.76±9.16% to 53.66%±8.17, p>0.05) (Fig 3E, 3F and 3G). Hence, VGLUT1 was the predominant VGLUT on DCX+ cells whilst VGLUT3 was predominant on GFAP+ cells at 7 dd.

Intriguingly, VGLUT1 and 3 were abundant in B3T+ cells at 3dd (14.0±3.2% and 8.2±2.1% respectively) whereas VGLUT2 was present only in 4.3±1.4% of cells (S5 Fig). Since B3T is expressed in a later stage of neuronal development [17], this suggests that all VGLUTs might be important during early differentiation stages of SVZ-derived NPCs while DCX is still highly expressed. The fact that VGLUT3 maintains high levels of expression at 7 dd in glial cells, compared to VGLUT1 and 2, which undergo a significant reduction in the same period, indicates that this transporter may play the most important role in glutamate vesicle loading in NPC derived glial cells.

### VGLUT1 and VGLUT2 inhibition increases neuronal differentiation

**Fig 4:**
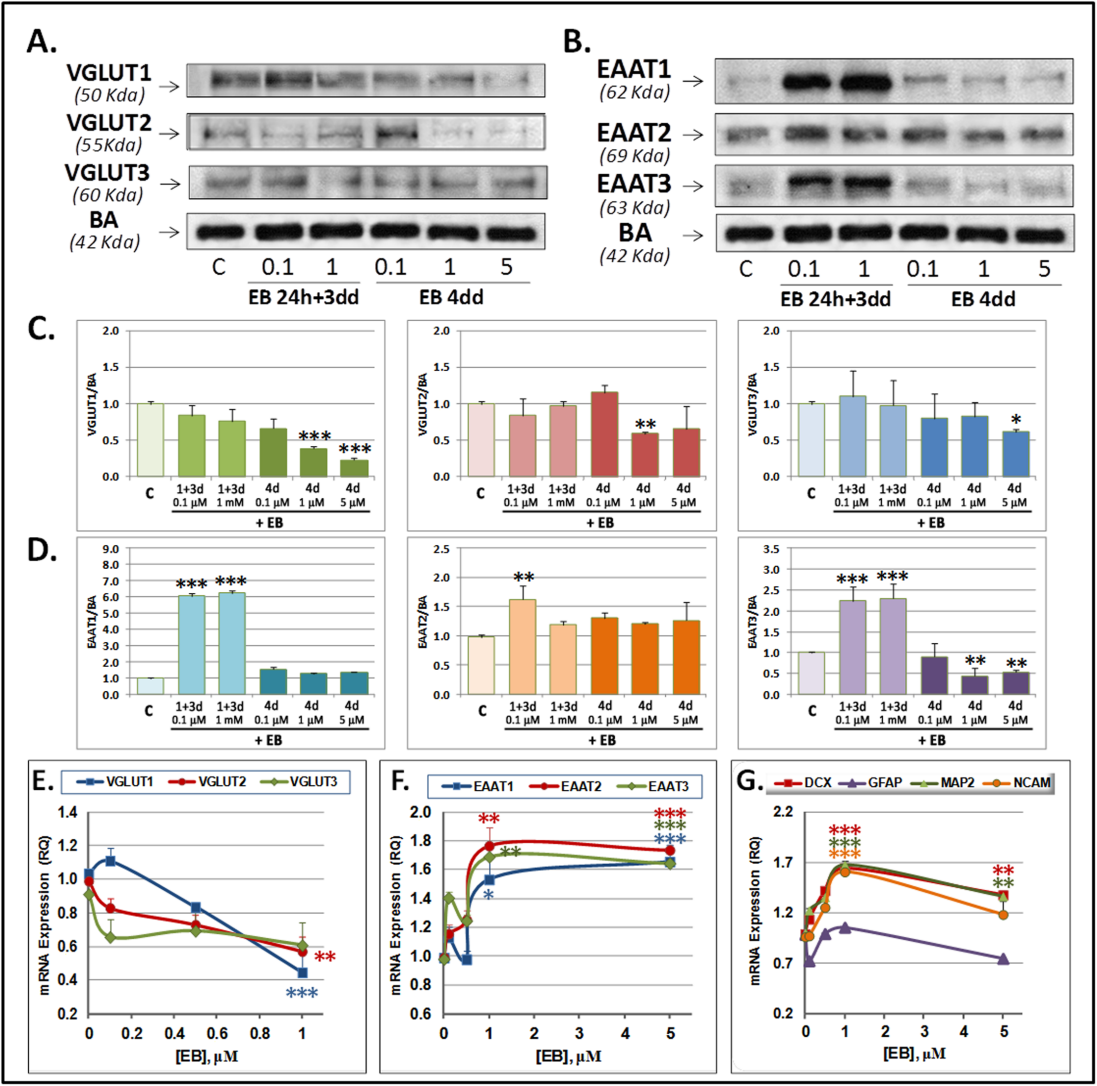
**Evans Blue (EB) effect on VGLUT and EAAT expression at the protein (A-D) and the mRNA level (E-F) and on mRNA expression of neurogenic markers (G) in SVZ NPCs in primary culture.** Cells were acutely (24 h EB + 3 dd) or chronically (4 dd) treated with EB at indicated concentrations and then analyzed by RTqPCR for mRNA expression. A-B) Representative Westerns blots of VGLUTs (A) and EAATs (B) and quantification normalized to β-actin (C-D). (E-G). Graphs show data of VGLUTs (E), EAATs (F) or DCX, GFAP, MAP2 and NCAM (G) mRNA expression. Data are means ± SEM of three experiments each one performed by duplicate in different cultures. Statistical significances against controls were performed by One Way ANOVA followed by Tukey post hoc test, when analysis of variance was significant. (*) P<0.05, (**) P<0.01 and (***) P<0,001.

To elucidate the involvement of VGLUTs in neurogenesis, we administered the competitive inhibitor of vesicular glutamate uptake Evans Blue (EB) [37], which blocks VGLUTs by interfering with their cation-binding sites [38]. Cells were acutely treated either for 24 h and then allowed to recover for 3 days, or continuously treated with increasing EB concentrations during 4 days. While acute EB treatment did not significantly alter VGLUT protein levels, chronic treatment significantly reduced all VGLUTs by more than 50% (Fig 4A and 4C). Continuous EB also reduced VGLUT mRNA expression (Fig 4E). Surprisingly, protein and mRNA levels of all EAATs were increased following acute but not chronic EB treatment, with the exception of EAAT3, which was mildly reduced at the protein level (Fig 4B, 4D and 4F). However, concentrations higher than 1 μM EB increased the mRNA levels of all EAATs (Fig 4F). Interestingly, mRNA levels of DCX, NCAM and MAP2 were significantly increased by 70% by 1 μM EB (Fig 4G). Of note, only 5 μM EB was able to reduce the number of cells on zone B of the cell culture (S1A and S6A Figs). Continuous EB exposure increased levels of Nestin, SOX2 and TLX, arguing against a possible reduction of glutamate transporter levels as a consequence of increased cell mortality (S6B Fig).

**Fig 5:**
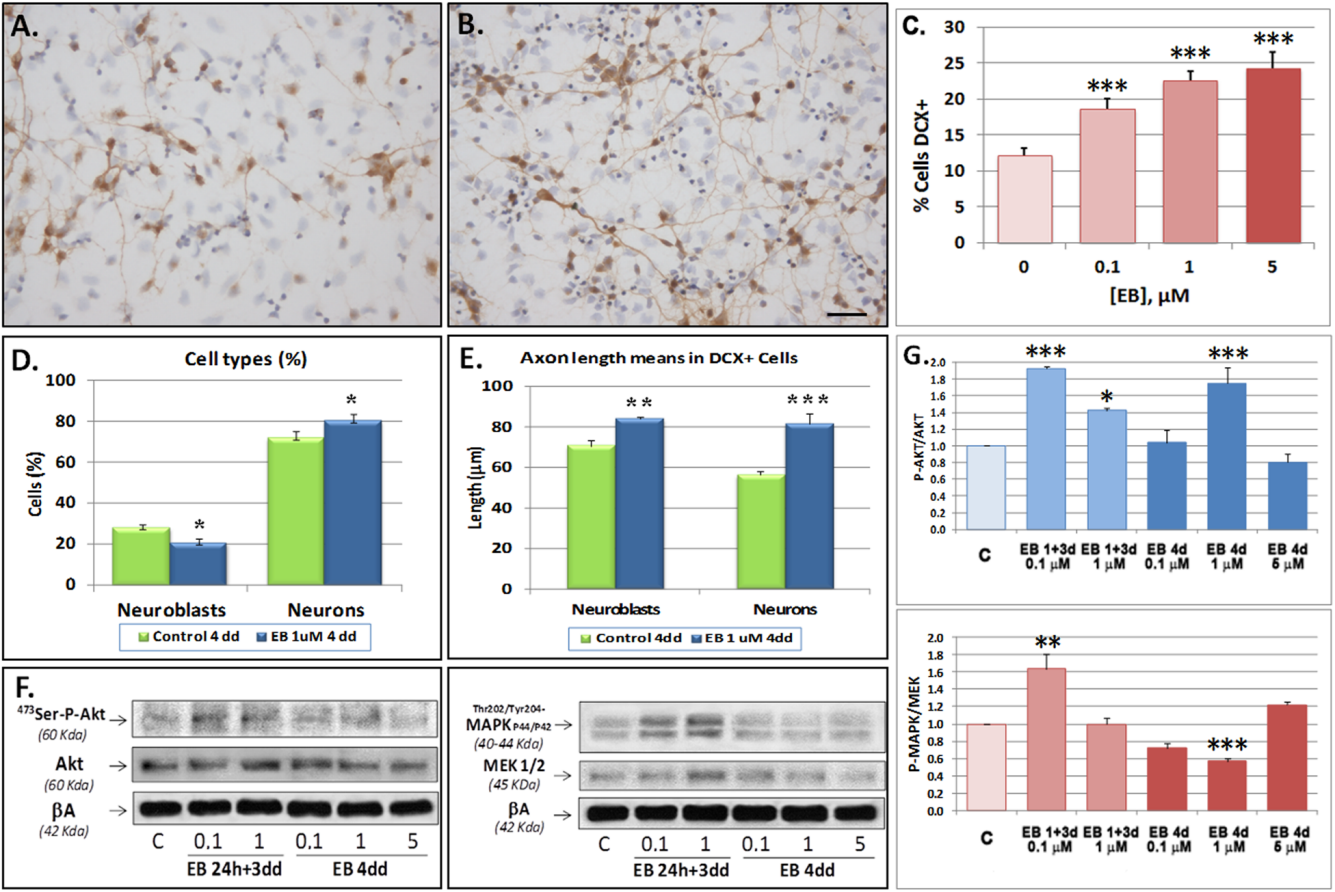
Evans Blue increases neuronal differentiation of SVZ-derived NPCs. **A-D**: NPCs immunocytochemical analysis of DCX+ immunolabelled cells. DCX+ cells are observed at 3 days of differentiation without EB (A) or with 1 μM chronic EB treatment (B). Note that DCX+ cells show more evident neurites than the control cells. C) Percentage of DCX+ cells increased in a concentration dependent manner under chronic treatment with EB at 3 days of differentiation. Chronic EB treatment significantly reduced the proportion of neuroblasts and increased the proportion of “neuronal-like” cells. D) Neuroblast and neuron percentages. E) Neurite length in neuroblasts and neurons (μm). Cells were characterized on area “B” (See S1 Fig). n = 900 cells/treatment. (F-G) Acute or chronic treatment of cells promoted ^473^Ser-Akt and phosphorylation and ^202Thr/204-Tyr^-MAPK P44/42 phosphorylation. Cells were acutely or chronically (4 dd) exposed to EB at the indicated concentrations and analyzed for P-AKT/AKT and P-MAPK/MEK. F) Representative Western Blot of P-AKT/AKT (left) or P-MAPK/MEK. G) Quantification of results for three different experiments. Statistical analysis was performed by One Way ANOVA, followed by Tukey post hoc test when analysis of variance was significant. Comparisons of percentages of neuroblasts or “neuronal-like” cells were done by Student-T test. (*) p<0.05, (**) p<0.01 and (***) p<0,001.

More detailed analyses using concentrations from 0.1 to 5 μM EB revealed a concentration dependent increase in the number of DCX+ cells (Fig 5A–5C), indicating that VGLUT1 and/or VGLUT2 inhibition promoted neuronal differentiation. In our cell cultures, some DCX+ cells indeed acquired a neuronal-like shape based on the presence of one characteristically longer neurite and several branches emerging from it, which was reminiscent of an axonal process, as opposed to the more usual bipolar shape of neuroblasts (S1B Fig). Quantitative analysis at 4dd revealed that EB at 1μM significantly decreased the number of neuroblasts from 28.09±4.26% to 20.92±5.49% but increased the number of neuronal like cells from 70.57±6.99% to 83.85±2.62% (Fig 5D). The length of neurites in neuroblasts and neuronal-like cells significantly increased from 70.57±2.85 μm to 83.85±1.07 μm and from 56.21±1.76 μm to 81.4±5.26 μm, respectively (Fig 5E).

Neuronal polarization is controlled by ^473^Ser-AKT phosphorylation, which in turn inhibits GSK3β thus activating microtubule polymerization and allowing axonal specification [39, 40]. Notably, EB treatment at 1μM for 4 days increased AKT phosphorylation by 1.75±0.19-fold whereas it decreased MAPK p44/42 phosphorylation to 0.57±0.03-fold (Fig 5F–5G). These results corroborated the notion that cell differentiation was induced by VGLUT downregulation.

## Discussion

During the past decade, glutamate and its receptors have been proven to play a critical role in the regulation of in neuroblast migration and survival along the glial tube and on NPC survival [17, 19, 29, 30], while it is also known that glutamate-mediated calcium currents are necessary for NPC neuronal differentiation of human NPCs [41]. However, a specific role of the glutamate transporters VGLUT and EAAT in the regulation of adult neurogenesis has so far not been documented. Previous work of our group showed that VGLUT1 and EAAT2 expression increases after focal cerebral ischemia, correlating positively with neurological damage, which suggests that VGLUT1 could influence brain remodeling and recovery by controlling the amount of glutamate that is loaded in presynaptic vesicles in the recovering brain. Additionally, VGLUT2 and 3 were also found in the iCC after stroke [10]. In agreement with our previous work, EAATs were found to be expressed by glial cells in the iCC [11]. Therefore, we wondered whether VGLUTs could be expressed in SVZ-derived NPCs and whether they play a role in neuronal differentiation. In order to test the role of glutamate transporters in neurogenesis, we characterized the expression of VGLUTs and EAATs in SVZ-derived NPC cultures at different stages of differentiation *in vitro*.

We found that the expression of VGLUT and EAAT in SVZ-derived NPCs increase throughout the differentiation process at both the mRNA and the protein level. VGLUT and EAAT expression correlated well with the expression of neuronal and glial differentiation markers, whilst there was an overall negative correlation with Nestin, showing that vesicular and membrane glutamate transporter expression is a hallmark of NPC differentiation. Furthermore, VGLUT1 and 2 are preferentially expressed by neuroblasts, whereas VGLUT3 is preferentially expressed by glial cells. Thus, VGLUT1 and 2 could be contributing mainly to neuronal differentiation whilst VGLUT3, in concert with EAAT2 (and to a lesser extent with EAAT1), could be contributing mainly to glial differentiation of NPCs *in vitro*. Importantly the stimulation of the P2Y4 receptor on embryonic stem cells was found to induce neuronal differentiation along with VGLUT expression, however VGLUT function was not evaluated in this study [42].

Pharmacological VGLUT inhibition with EB increased mRNA expression of the neurogenic markers DCX, B3T and MAP2, the number of NPCs expressing DCX and the differentiation from neuroblasts to neurons as judged by increased axonal length, together with activation of pAKT, which through inhibition of GSK3β can regulate axonal and dendritic specification [43], thereby suggesting that VGLUTs activity averted the entry of NPCs into neuronal differentiation. Importantly, increased signalling through the PI3K/Akt/NF-κB pathway and GSK3β inhibition has been shown to increase EAAT expression [44–46]. Thus, it could be speculated that EB promoted the EAAT expression under acute stimulation by a mechanism that is then silenced during continuous stimulation. On the other hand a VGLUT repressor might have been expressed under continuous EB stimulation through this pathway. Indeed when exposed to EB 1μM, VGLUTs and EAATs showed a differential regulation (Fig 4). Of note, the activation of AKT could drive mTOR-Akt-NF-κB cascade activation which has been shown to play critical roles to up-regulate GLT-1 after oxygen glucose deprivation [44].

EB is a very potent inhibitor of VGLUT activity [37], however a pharmacological approach often has the problem of lack of specificity. EB is also impermeable to the blood-brain-barrier, thus limiting the continuation of these studies *in vivo*. Further studies using siRNA to specifically delete the different isoforms of VGLUTs, or other VGLUT inhibitors such as Chicago Sky Blue 6, which has been used *in vivo* to study the contribution of VLGUT2 to neuropathic pain [46], will further enhance our understanding of the role of each of these transporters on neuronal differentiation both *in vivo* and *in vitro*. However, a study of how adult neurogenesis could be altered in adult animals lacking VGLUTs seems unlikely since VGLUT1 -/- animals show lethality after the third week of age [47] whilst VGLUT2 -/- die right after birth [48]. Thus, strategies must be developed to selectively delete each VGLUT on SVZ-NPCs so that their contribution to neurogenesis in health and disease can be further confirmed.

Although VGLUTs have been clearly shown to influence plasticity in a wide variety of contexts of health and disease [48, 49], to the best of our knowledge, this is the first work showing an active role of presynaptic proteins in the process of NPC differentiation *in vitro*. Nevertheless the involvement of other proteins of the SNARE complex, necessary for proper vesicle docking [50], remain to be elucidated in this context. Our data indicate that VGLUT-mediated glutamate release from SVZ-derived neuroblasts or astrocytes provides an autocrine signal that controls neuroblast migration and differentiation. This is of the utmost importance in the context of a brain trauma in which glutamate is highly dysregulated, such as stroke, a condition in which VGLUT regulation and function has been barely characterized [10]. The inhibitory control exerted by VGLUT activity on neural differentiation could thus regulate the timing at which the cell becomes a neuron, allowing for correct migration and synaptic integration within the surviving neuronal network by preventing the cell differentiating at the wrong location.

## Conclusions

**Fig 6:**
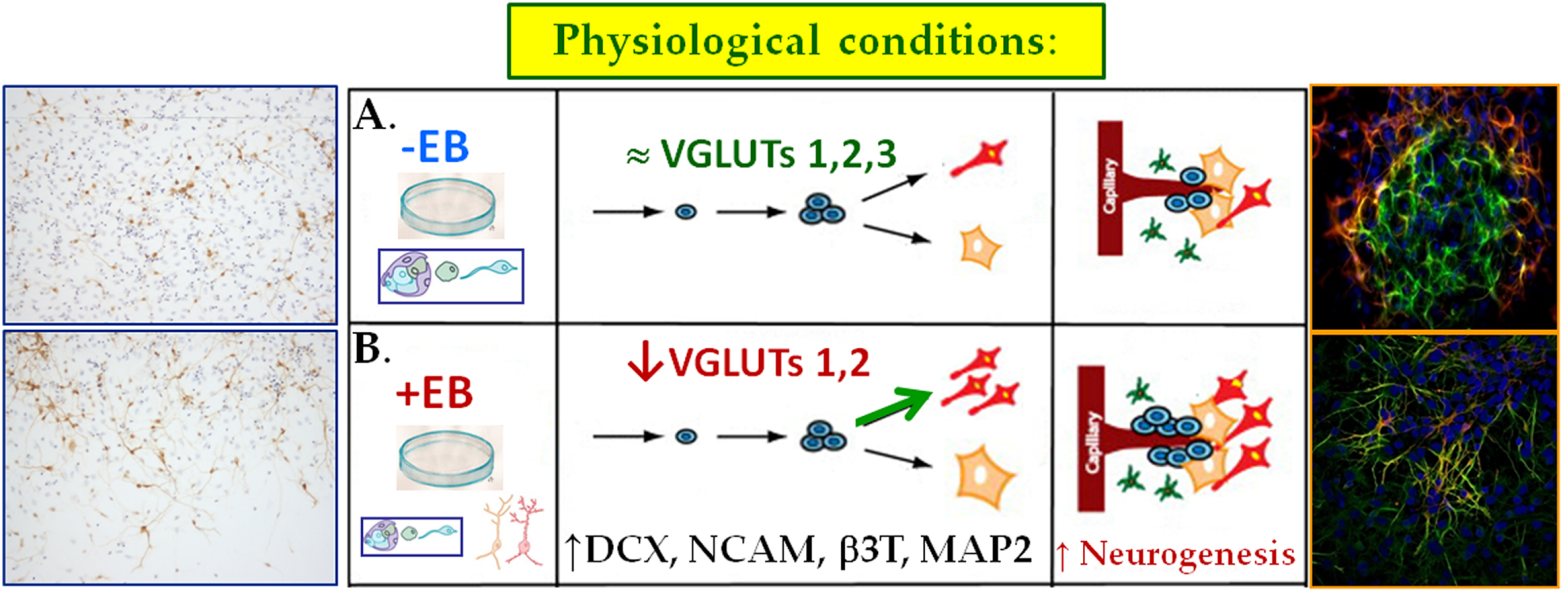
Proposed model for the role of VGLUTs in neuronal differentiation of cultured SVZ-derived neural precursor cells. Under physiological conditions, VGLUT1-2 inhibition, and thus decreased glutamate exocytosis, may promote neuronal differentiation of NPCs.

In short, in the light to our results we propose that (Fig 6):

- NPCs express VGLUT1-3 and EAAT1-3 both at the mRNA and protein level.
- Their expression augments throughout NSC differentiation.
- The inhibition of VGLUT expression increases adult NSC neuronal differentiation.
- Under physiological conditions, VGLUT1-3 inhibition promotes neuronal differentiation and migration of NPCs.

## Supporting information

S1 Fig SVZ neurospheres after 3 days of differentiation in primary culture. **A)** Organization of NPCs in culture: Cells migrate from zone A (center) to zone B (periphery) where they acquire their final phenotype. Double immunocytochemistry of nestin (red) and VGLUT2 (green) at three days of differentiation. Dapi (blue) was used for nuclear staining. **B)** Comparison between a neuroblast (★) and a neuronal-like cell or “proto-neuron” (▲), both expressing DCX. Asterisk: a typical bipolar neuroblast cell. Arrow head: A cell that we have considered as a proto-neuron. Of note the neuroblast has two large projections from which an axon is indistinguishable, and almost no collateral branching. The proto-neuron has a characteristic primary axonal like extension (bracket) with several primary and secondary collateral branches and primary dendrites emerging from the cell body (arrow). Scale bar = 7 μm.
(TIF)

S2 Fig Neuroblasts and proto-neurons express MAP2 and DCX at 3dd. A) MAP2 shows an expression pattern with highest expression on the axonal shaft (*) while DCX is ditributed all allong the cell. B-D) MAP2 allows the detection of the axonal fiber (B) while DCX allows the detection of the axon, axonal tip (C; arrow head) and collateral fibers (C; arrows), as evidenced in the merge image (D). This allows a better estimation of axonal length on DCX+ cells. E) Protoneurons also present higher expression of MAP2 on the basis of the axonal shaft (*). F-H) MAP2 shows faint expression on collaterals (F; arrows) while DCX labelling clearly stains not only the axonal shaft but also axonal collaterals (G; arrows), as shown in the merged image (H). Scale bar: 20 μM (A, E); 10 μM (B-D; F-H).
(TIF)

S3 Fig Correlation analysis between glutamate transporter (VGLUTs and EAATs) mRNA expressions in SVZ NPCs and mRNA expression of specific markers of neurogenesis. Linear regression analysis of correlations between: A) VGLUT1 and VGLUT2 vs. each other and vs. NEST or GFAP (B-E). F-J. Correlations between VGLUT3 and different neurogenic markers. K) Correlations between EAAT2 and VGLUT3 each other and between EAAT2 and indicated neurogenic markers (L-O). Straight line equations, correlation coefficients (r) and statistical significances of regression analyses are indicated in each plot. Regression analysis and statistics were performed by the Spearman Rank Order Correlation Test.* p<0.05; ** p<0.01 and *** p<0.001.
(TIF)

S4 Fig Observation of VGLUT + vesicles in cultures of differentiated CPNs. A representative image of the punctate pattern found for all VGLUTs proteins in our culture conditions is shown. VGLUTs can be found in different cell populations (compare asterisk vs. arrow). In this case the colocalization of a VGLUT with DCX, is shown, thus demonstrating that VGLUTs are expressed in neuroblasts
(TIF)

S5 Fig Coexpression of VGLUT1-3 and B3T in NPCs at 3 days of differentiation. A-B) VGLUT1, B3T, DAPI and merge images at 3 days of cell differentiation. C-D) VGLUT2. E-F) VGLUT3. Quantification was performed by counting of double labelled cells with respect to the total cells in the field as determined by DAPI staining. Data are expressed as mean ± SEM. 150–200 cells per image were analyzed; n = 10 images from 2 different cultures. Scale bar = 20 μm.
(TIF)

S6 Fig EB treatment does not alter cell survival signifficantly but slightly increases proliferation. A) Total cell number was estimated by counting hematoxylin dyed nuclei on zone B of the cell culture. Only 5 μM EB was able to significantly reduce the number of nuclei (259±21 cells/field versus 377±20 cells/field for the control; n = 20 fields) in zone B of the cell culture. B) Nestin, SOX2 and TLX were increased after prolonged incubation with different concentrations of EB suggesting that EB may increase proliferation even in the absence of growth factors. Data are means ± SEM of three experiments each one performed by duplicate in different cultures. Statistical significances against controls were performed by One Way ANOVA followed by Tukey post hoc test, when analysis of variance was significant. (*) P<0.05, (**) P<0.01 and (***) P<0,001.
(TIF)
