# Supplementary figures and images for: Vesicular glutamate transporters play a role in neuronal differentiation of cultured SVZ-derived neural precursor cells
Source: PLoS One. 2017 May 11;12(5):e0177069. doi: 10.1371/journal.pone.0177069 (PMC5426660; doi:10.1371/journal.pone.0177069)

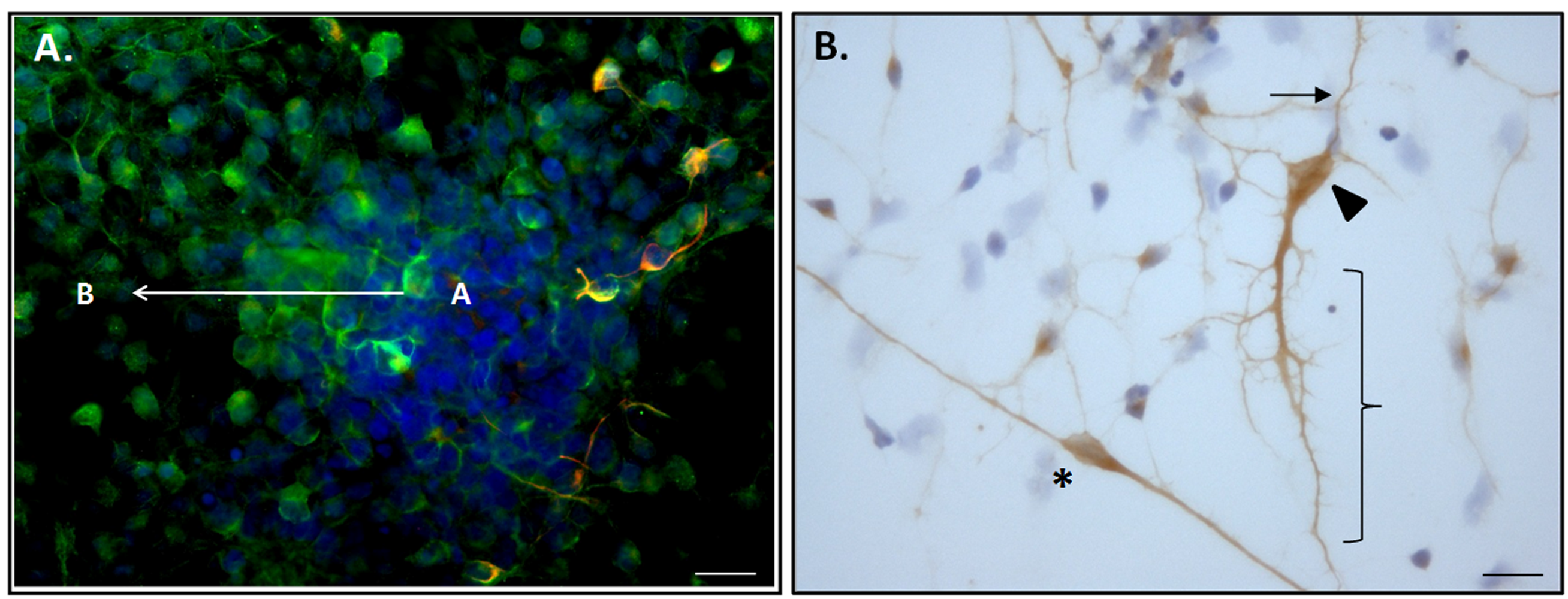

Supplement: S1 Fig — A) Organization of NPCs in culture: Cells migrate from zone A (center) to zone B (periphery) where they acquire their final phenotype. Double immunocytochemistry of nestin (red) and VGLUT2 (green) at three days of differentiation. Dapi (blue) was used for nuclear staining. B) Comparison between a neuroblast (★) and a neuronal-like cell or “proto-neuron” (▲), both expressing DCX. Asterisk: a typical bipolar neuroblast cell. Arrow head: A cell that we have considered as a proto-neuron. Of note the neuroblast has two large projections from which an axon is indistinguishable, and almost no collateral branching. The proto-neuron has a characteristic primary axonal like extension (bracket) with several primary and secondary collateral branches and primary dendrites emerging from the cell body (arrow). Scale bar = 7 μm. (TIF) [file pone.0177069.s001.tif]

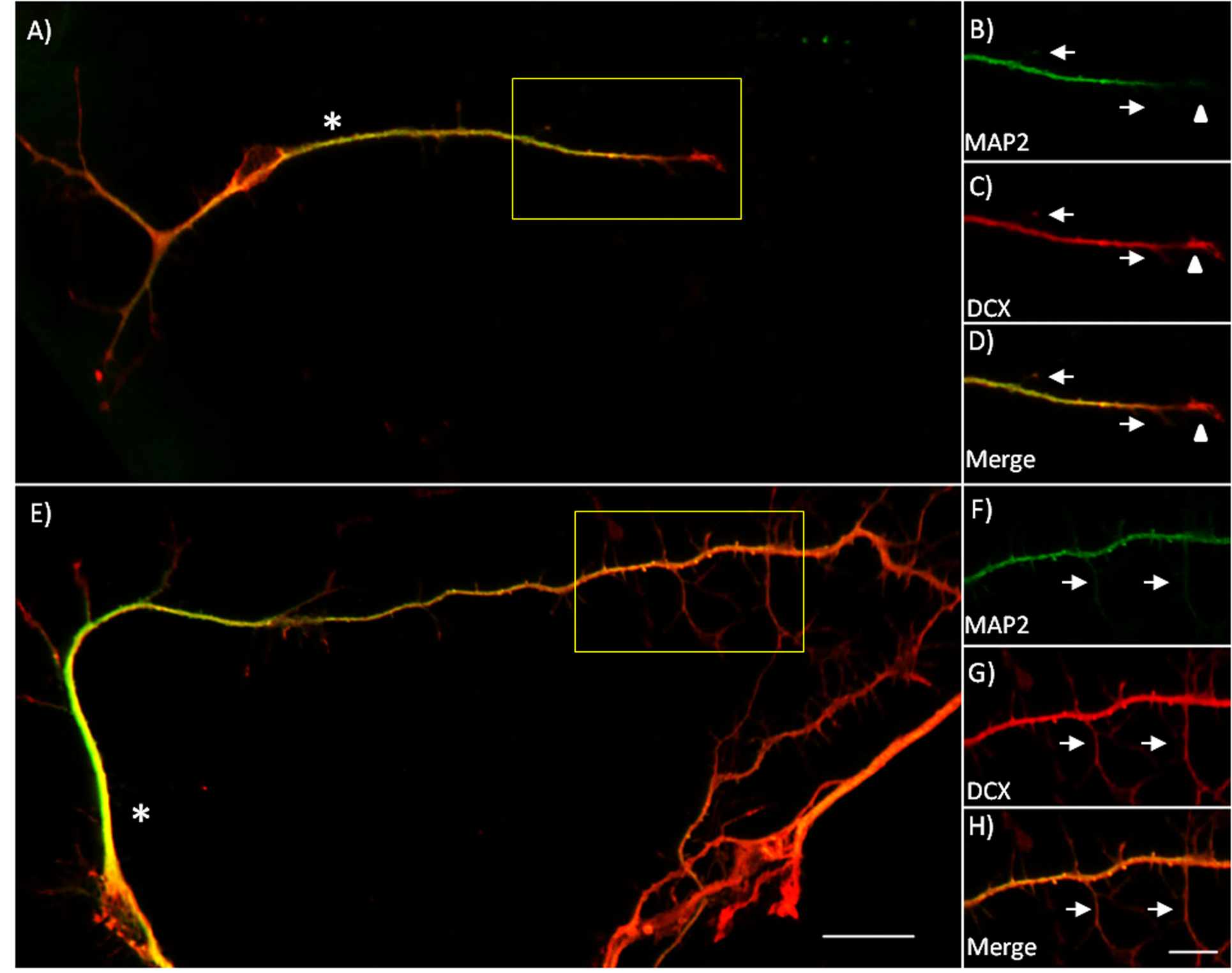

Supplement: S2 Fig — A) MAP2 shows an expression pattern with highest expression on the axonal shaft (*) while DCX is ditributed all allong the cell. B-D) MAP2 allows the detection of the axonal fiber (B) while DCX allows the detection of the axon, axonal tip (C; arrow head) and collateral fibers (C; arrows), as evidenced in the merge image (D). This allows a better estimation of axonal length on DCX+ cells. E) Protoneurons also present higher expression of MAP2 on the basis of the axonal shaft (*). F-H) MAP2 shows faint expression on collaterals (F; arrows) while DCX labelling clearly stains not only the axonal shaft but also axonal collaterals (G; arrows), as shown in the merged image (H). Scale bar: 20 μM (A, E); 10 μM (B-D; F-H). (TIF) [file pone.0177069.s002.tif]

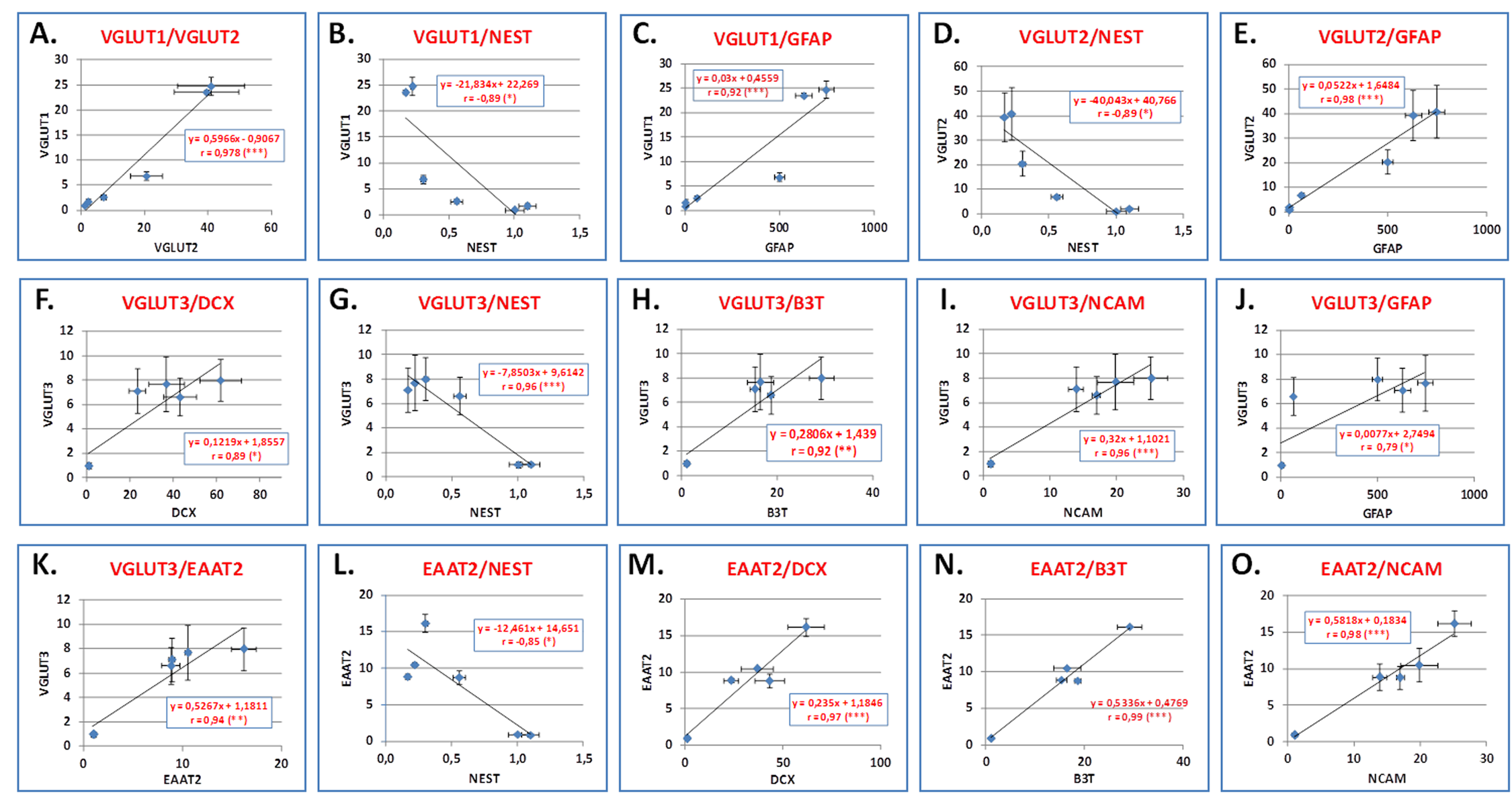

Supplement: S3 Fig — Linear regression analysis of correlations between: A) VGLUT1 and VGLUT2 vs. each other and vs. NEST or GFAP (B-E). F-J. Correlations between VGLUT3 and different neurogenic markers. K) Correlations between EAAT2 and VGLUT3 each other and between EAAT2 and indicated neurogenic markers (L-O). Straight line equations, correlation coefficients (r) and statistical significances of regression analyses are indicated in each plot. Regression analysis and statistics were performed by the Spearman Rank Order Correlation Test.* p<0.05; ** p<0.01 and *** p<0.001. (TIF) [file pone.0177069.s003.tif]

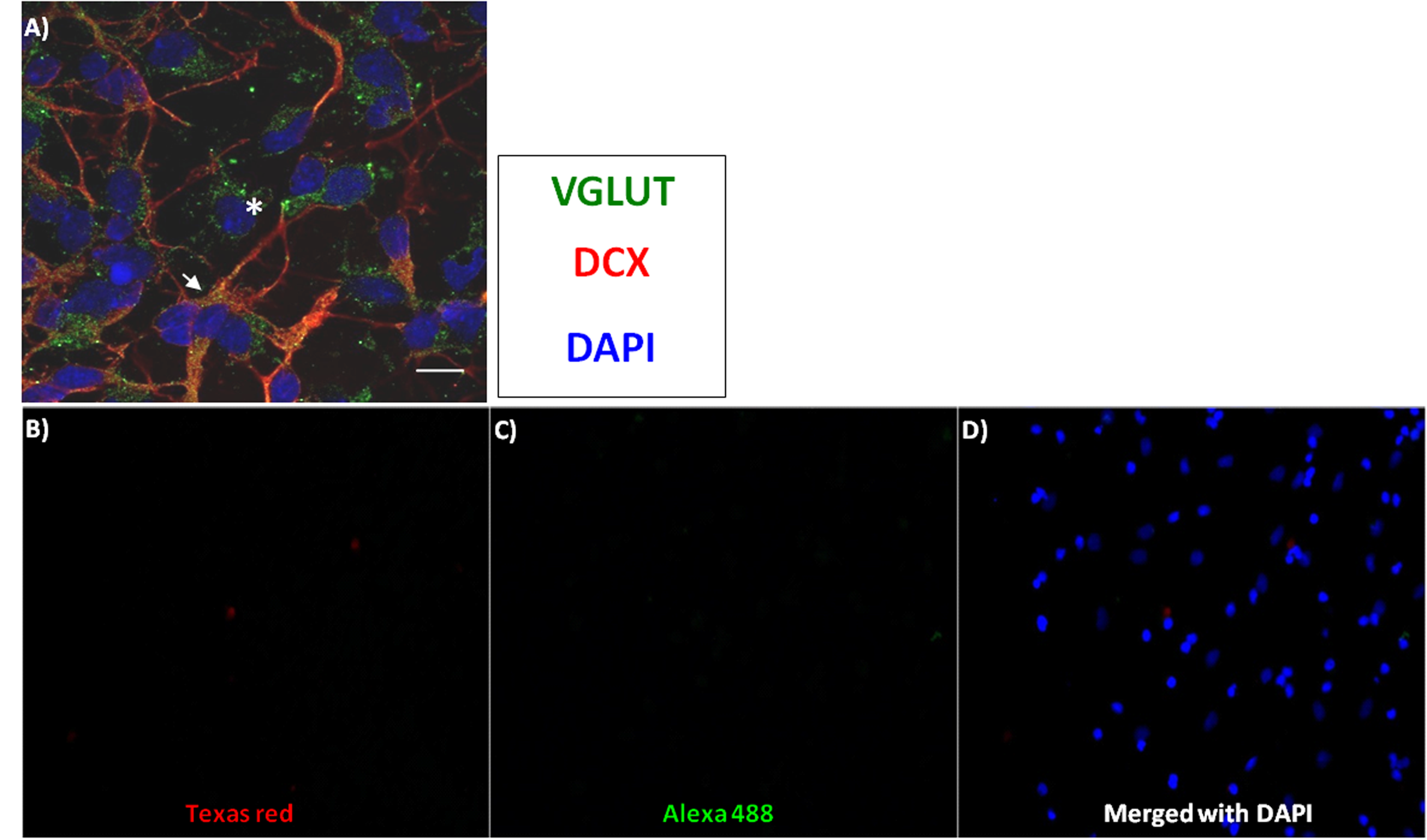

Supplement: S4 Fig — A representative image of the punctate pattern found for all VGLUTs proteins in our culture conditions is shown. VGLUTs can be found in different cell populations (compare asterisk vs. arrow). In this case the colocalization of a VGLUT with DCX, is shown, thus demonstrating that VGLUTs are expressed in neuroblasts (TIF) [file pone.0177069.s004.tif]

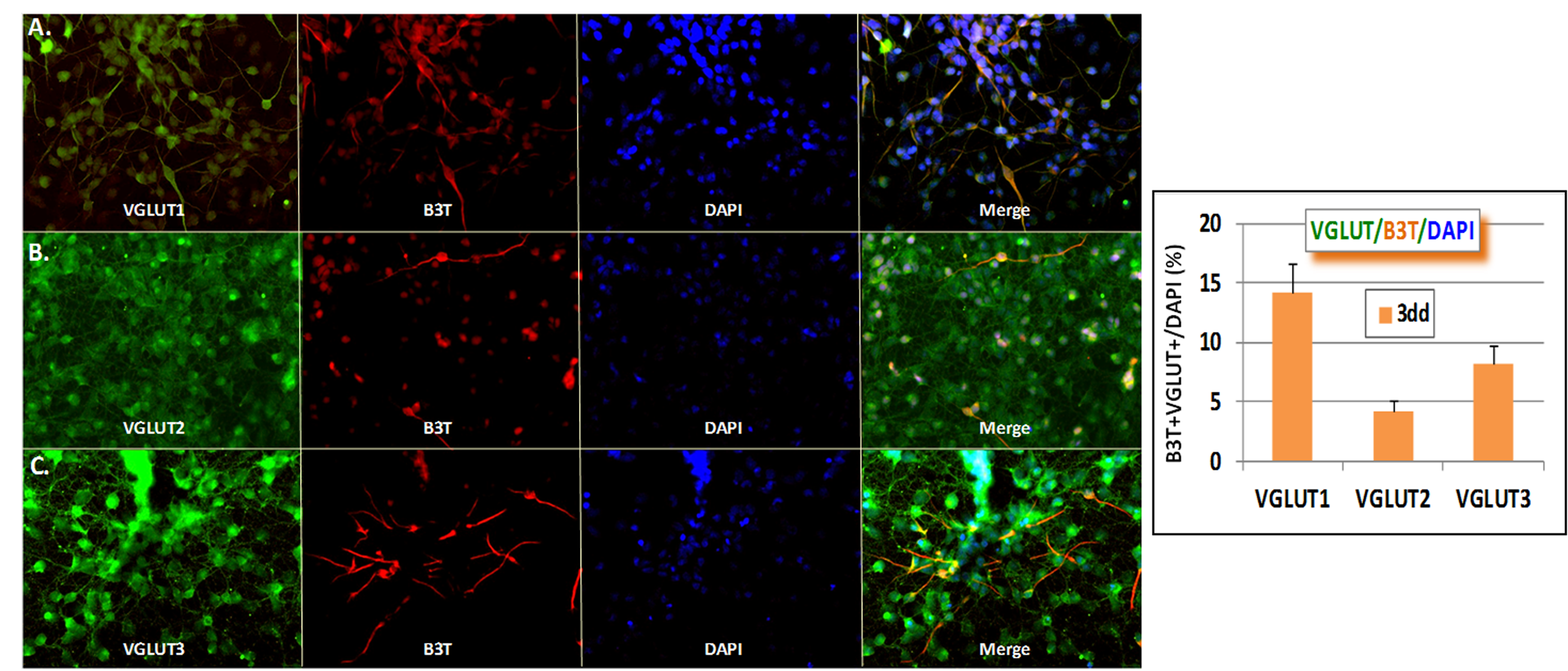

Supplement: S5 Fig — A-B) VGLUT1, B3T, DAPI and merge images at 3 days of cell differentiation. C-D) VGLUT2. E-F) VGLUT3. Quantification was performed by counting of double labelled cells with respect to the total cells in the field as determined by DAPI staining. Data are expressed as mean ± SEM. 150–200 cells per image were analyzed; n = 10 images from 2 different cultures. Scale bar = 20 μm. (TIF) [file pone.0177069.s005.tif]

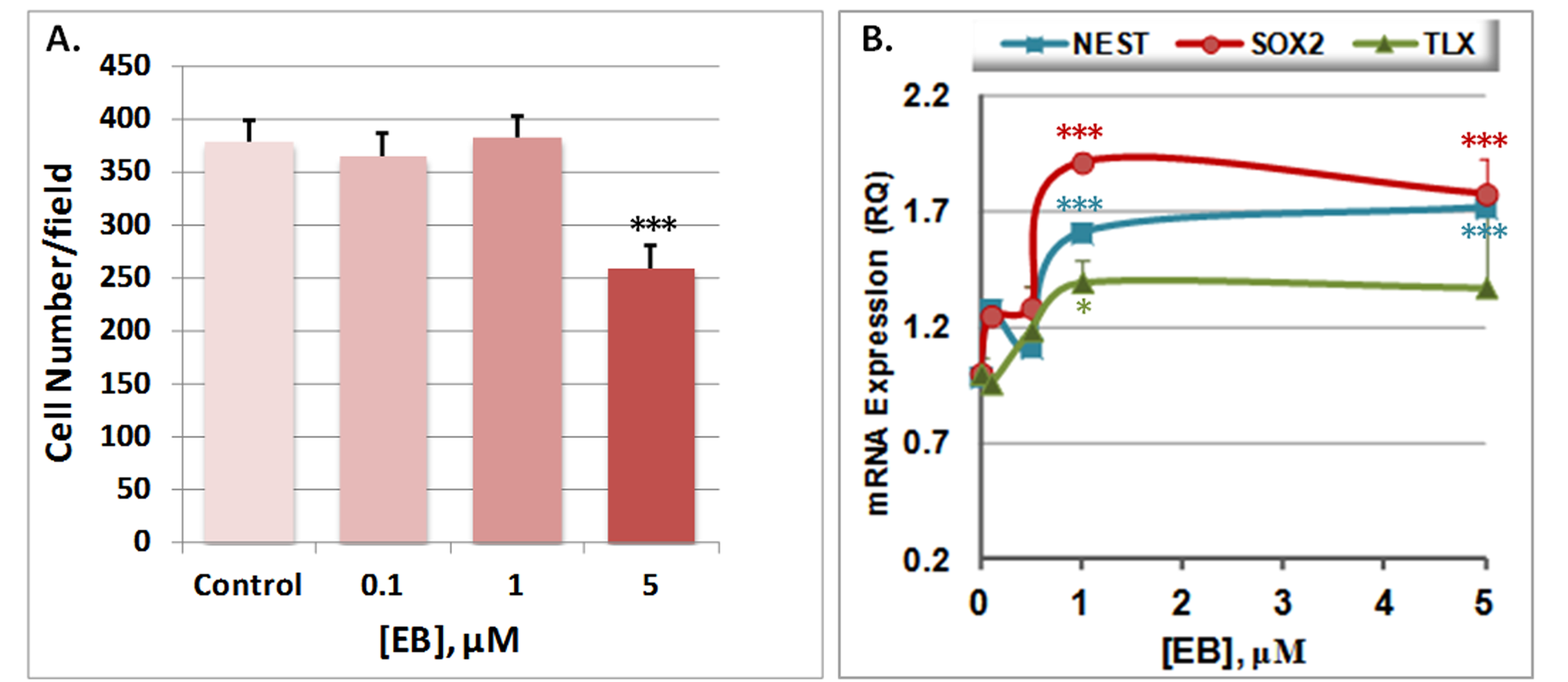

Supplement: S6 Fig — A) Total cell number was estimated by counting hematoxylin dyed nuclei on zone B of the cell culture. Only 5 μM EB was able to significantly reduce the number of nuclei (259±21 cells/field versus 377±20 cells/field for the control; n = 20 fields) in zone B of the cell culture. B) Nestin, SOX2 and TLX were increased after prolonged incubation with different concentrations of EB suggesting that EB may increase proliferation even in the absence of growth factors. Data are means ± SEM of three experiments each one performed by duplicate in different cultures. Statistical significances against controls were performed by One Way ANOVA followed by Tukey post hoc test, when analysis of variance was significant. (*) P<0.05, (**) P<0.01 and (***) P<0,001. (TIF) [file pone.0177069.s006.tif]
